# Determination of the minimal level of neutralizing antibodies elicited following vaccination able to protect rabbits against virulent cowpox virus

**DOI:** 10.3389/fimmu.2025.1640056

**Published:** 2025-07-21

**Authors:** Muratbay Mambetaliyev, Alina Alieva, Yergali Abduraimov, Aralbek Rsaliyev, Kuandyk Zhugunissov

**Affiliations:** ^1^ Laboratory of Microorganism Collection, Research Institute for Biological Safety Problems, Guardeyskiy, Kazakhstan; ^2^ Department of Biological Safety, National Holding "QazBioPharm", Astana, Kazakhstan

**Keywords:** virus, cowpox, immunity, antibody, virus neutralization test, challenge infection

## Abstract

**Background:**

Serological assessment of antibody levels is a crucial measure of immunity in vaccinated animals. Establishing the level of antibodies considered protective is essential for vaccine standardization and evaluation of efficacy. The virus neutralization test (VNT), recognized as the gold standard for detecting virus-specific antibodies able to neutralize virus.

**Methods:**

This study evaluated the effect of viral dose on the detection of humoral immune responses in rabbits vaccinated with a cowpox virus-based vaccine. Blood serum samples were collected on days 14, 21, and 28 post-vaccination. VNT was conducted using viral doses of 100, 50, 25, and 10 TCID_50_. Additionally, the infectious dose 50 (ID_50_) of the challenge virus was determined based on the induction of skin necrosis in 50% of infected animals. This dose (316 ID_50_ per 0.1 mL) was then used to challenge vaccinated rabbits in order to determine the protective antibody titer threshold.

**Results:**

Lower viral doses (25 and 10 TCID_50_) demonstrated higher sensitivity, with neutralizing antibody titers detected at 1:16 and above, significantly exceeding those obtained using 50 and 100 TCID_50_. Based on these findings, 25 TCID_50_ was selected as the optimal dose for future VNT. Following cowpox virus challenge, rabbits with neutralizing titers ≥1:16 were protected from skin necrosis, while non-immunized animals developed characteristic lesions.

**Conclusion:**

These results suggest that a low-dose (25 TCID_50_) VNT improves the sensitivity and that a titer of 1:16 can be considered a protective threshold. This approach provides a reliable laboratory model for assessing the immunogenicity and efficacy of cowpox virus vaccines. The results obtained in this study allow for an objective assessment of the immunity elicited from a cowpox vaccine using a laboratory model.

## Introduction

Cowpox virus (CPXV) is an infectious agent belonging to the genus Orthopoxvirus within the family Poxviridae (1–3). It is a zoonotic virus with a broad host range, affecting both livestock and humans (2–4). Wild and predatory rodents serve as the primary reservoirs and potential vectors of CPXV transmission (5–7).

Despite its historical significance and zoonotic potential, CPXV remains insufficiently characterized. Existing literature on orthopoxviruses predominantly focuses on variola virus (the causative agent of smallpox) and vaccinia virus, often overlooking CPXV as a distinct nosological entity (8, 9). Consequently, data on the biological and physicochemical properties of CPXV, including its genomic structure and protein composition, remain limited.

The human population currently exhibits low to no immunity against orthopoxvirus infections, including smallpox, monkeypox, cowpox, and buffalopox (7). In recent years, a growing number of orthopoxvirus outbreaks have been reported in both humans and animals across various regions of the world (10). Vaccination remains the primary strategy for preventing these infections; however, questions regarding vaccine efficacy and the determination of protective antibody titers are yet to be fully addressed.

Serological assays play a central role in evaluating vaccine-induced immunity. The antibody level in blood serum is a key indicator of the humoral immune response. Determining the level of antibody titers which are considered to be protective is particularly important to determine vaccine efficacy. Among the available methods, enzyme-linked immunosorbent assay (ELISA) and the virus neutralization test (VNT) are commonly employed. While ELISA offers high-throughput screening, VNT remains the gold standard due to its ability to quantify functional neutralizing antibodies and the high specificity of the assay (11, 12). However, VNT protocols must be carefully optimized for each virus to ensure accurate results.

At the Research Institute for Biological Safety Problems, a CPXV vaccine was developed using a live attenuated orthopoxvirus cowpox virus strain CP-65K. Nevertheless, difficulties emerged in evaluating the vaccine’s capacity to induce humoral immunity. Specifically, the immune response in vaccinated animals could not be reliably confirmed using commercially available ELISA kits or standard neutralization assays. Despite these limitations, vaccinated animals were protected from challenge infection with a virulent CPXV strain (unpublished data), suggesting the presence of a protective immune response which could not be detected using the existing diagnostic tests.

The use of challenge infection models – where vaccinated animals are exposed to virulent viral strains – remains the most appropriate method to demonstrate vaccine efficacy. However, such experiments require high-level biosafety (ABSL-3) facilities, access to suitable laboratory animals, and significant financial and logistical resources. These constraints limit their routine application but underscore their value as a benchmark method for protective efficacy evaluation.

Given these challenges, there is a clear need to develop neutralization assay parameters and establish a reliable protective antibody threshold. While cellular immunity is known to play a critical role in the control of orthopoxvirus infections (13), its assessment involves complex and costly methodologies that are not easily integrated into routine research. Therefore, evaluating antibody levels remains the most practical and scalable approach for monitoring vaccine-induced protection.

The objective of this study was to optimize the virus neutralization assay with respect to the dose of virus used in the assay to be able to assess antibody titers in rabbit serum following CPXV vaccination. In addition, the determination of the antibody titers which are considered to be protective were established to evaluate the vaccine’s protective efficacy in a rabbit model.

## Materials and methods

### Cowpox virus strain and vaccine

The attenuated cowpox virus strain “CP-65K” was used as the vaccine candidate in this study. This strain was derived through 65 serial passages, including 15 passages in embryonated chicken eggs and 50 passages in lamb kidney cell culture. The resulting viral preparation had an infectious titer of (6.50 ± 0.08) log_10_ 50% Tissue Culture Infectious Dose (TCID_50_)/mL.

The virulent cowpox virus strain “Cowpox-CAM” was used as the challenge virus, with an infectious titer of (4.50 ± 0.08) log_10_ TCID_50_/mL.

### Animals and bioethics

Non-pedigree (mixed-breed) rabbits weighing between 2.5 and 3.5 kg with an initial body temperature of 38.5–39.0 °C were used as the laboratory model. Prior to the experiments, the animals were maintained under quarantine conditions for a period of one month. All procedures were conducted in an ABSL-3 (Animal Biosafety Level 3) facility equipped with HEPA filters at both the intake and exhaust airlocks, a pass-through autoclave, and a localized sanitation entry system with a shower. Animal housing and feeding were carried out in accordance with established guidelines (14).

All animal procedures were conducted in accordance with the Law on Responsible Treatment of Animals (Law No. 97-VII ZRK, Republic of Kazakhstan, December 30, 2021) and other applicable guidelines. The Bioethics Committee of the Research Institute for Biological Safety Problems approved the study protocols under permit No. 1001/023 prior to the commencement of the research. Institutional codes, standard operating procedures, and animal care guidelines were strictly followed throughout the entire study.

### Study design

The study design consisted of three stages. In the first stage, the virus dose for the VNT was optimized. For this purpose, the neutralization assay was performed using the method described below, with viral doses of 10, 25, 50, and 100 TCID_50_. The most effective dose among these was selected for subsequent experiments.

In the second stage, the standardize of the dose of challenge virus was determined using a 50% infectious dose (ID_50_), for evaluating the protective efficacy of the vaccine in immunized animals. The challenge virus strain was titrated using tenfold serial dilutions ranging from 10^-^¹ to 10^-6^. Each dilution was administered intradermally to four rabbits (n = 4 per dilution group). Clinical monitoring was performed daily, including measurement of body temperature, observation of the general health status, and recording of pathological changes at the injection sites.

The presence of specific cutaneous lesions at inoculation site was used as the primary criterion for infection. The virus titer was calculated using the Reed and Muench method, which estimates the 50% endpoint based on the proportion of animals showing infection at each dilution level (Reed & Muench, 1938) (15).

In the third stage, vaccine evaluation was performed using a group of rabbits (n = 20) was immunized subcutaneously with a dose of 10,000 TCID_50_. Prior to vaccine administration, the skin at the injection site was disinfected with 70% ethanol. Blood serum samples were collected on days 7, 14, 21, and 28 post-vaccination to assess the levels of virus-neutralizing antibodies (VNA) against the CPXV using a VNT.

Twenty-eight days following vaccination rabbits were challenged intradermally with the virulent CPXV Cowpox-CAM strain at a dose of 316 ID_50_/0.1 mL, to evaluate the protective efficacy of the vaccine. Clinical observation of the infected animals was conducted daily for 21 days and included monitoring of body temperature and clinical signs. The pathogenicity of the virus was assessed based on the appearance of cutaneous reactions (necrosis) at the inoculation site.

At the end of the observation period, a comparative analysis of the virus neutralization test results and the outcomes of the challenge infection was performed to determine the correlation between these parameters.

### Virus neutralization test

The VNT was conducted following the protocol reported by Manenti et al. (16), with necessary modifications. Serum samples were heat-inactivated at 56°C for 1 hour prior to testing. Two-fold serial dilutions of the serum, ranging from 1:2 to 1:256, were prepared and mixed with an equal volume of CPXV solution containing 25 TCID50. The serum-virus mixture was incubated for 1 hour at 37°C in a humidified atmosphere containing 5% CO2.

Following the incubation period, 100 μL of the serum-virus mixture was transferred to 96-well plates seeded with lamb kidney cells. Plates were incubated at 37°C in a humidified atmosphere containing 5% CO2 for 7–10 days. During this time, plates were inspected daily using an inverted optical microscope to evaluate the cytopathic effect (CPE) at each dilution point.

The neutralization titer was determined as the highest serum dilution that completely inhibited the CPE, indicating effective neutralization of the cowpox virus. Results were recorded as the reciprocal of this dilution and expressed in terms of neutralizing antibody titers.

### Determination of the 50% infectious dose of the challenge virus

The experiment was conducted on 24 rabbits weighing between 2.5 and 3.5 kg. The hair on the lateral surface of the body at the sites of virus administration was shaved prior to inoculation. The challenge virus strain was titrated using serial tenfold dilutions from 10–^1^ to 10^-6^. Each dilution was administered intradermally to four rabbits (n = 4 per dilution) in a volume of 0.1 mL per injection site. Clinical observation of the inoculated animals was carried out for 21 days. The infectious dose was determined based on the development of necrotic lesions at the injection sites. The virus titer was calculated according to the method of Reed and Muench (Reed L.J., Muench H.A.) (15).

### Statistical analysis

Statistical analysis of the experimental results was performed using GraphPad Prism 10 (GraphPad Software, Inc., La Jolla, CA, USA). Mean values and standard deviations of the obtained quantitative indicators were calculated.

The relationship between virus-neutralizing antibody titers and the level of protection following challenge infection was assessed using descriptive statistics and graphical methods. The percentage of infected animals at each virus dilution was plotted against the corresponding logarithmic dilution values to generate a dose–response curve. The 50% infectious dose (ID_50_) was estimated by interpolation using the Reed and Muench method. The confidence interval between virus doses used in the neutralization assay was determined using two-way analysis of variance (2-way ANOVA). Data processing was carried out using Microsoft Excel and dedicated statistical software for biological analysis.

## Results

### Optimization of the virus dose for the neutralization test

In our previous studies (17, 18), virus neutralizing antibodies in animals vaccinated against cowpox were not reliably detected when using standard virus doses (100 or 1000 TCID_50_) in the VNT. Therefore, to increase the sensitivity of the VNT, the virus dose used in the VNT was titrated and optimized.

**Figure 1:**
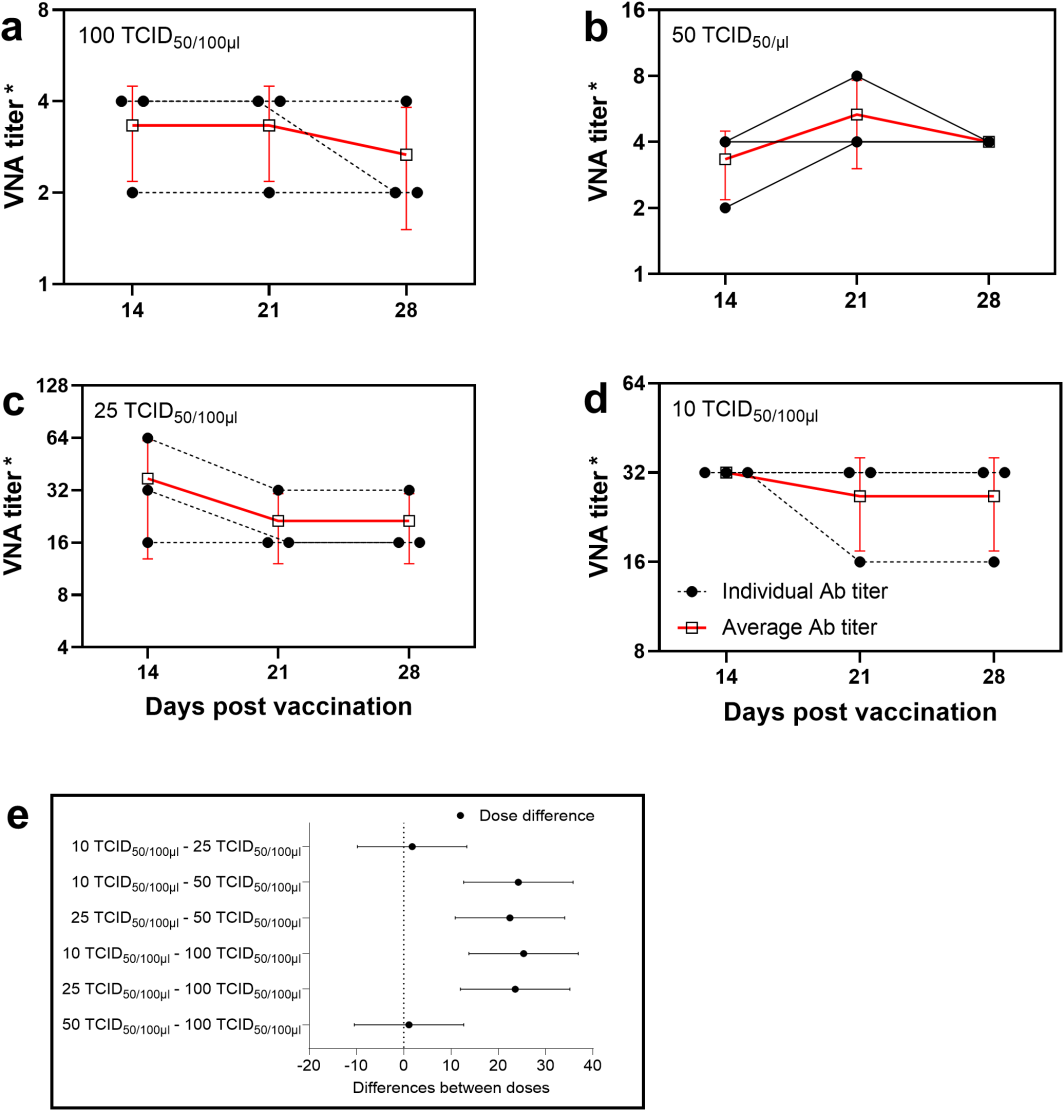
Determination of the optimal virus dose for neutralization assay. Antibody titers in rabbit sera determined by VNT using different virus doses: **(a)** 100 TCID_50_, **(b)** 50 TCID_50_, **(c)** 25 TCID_50_, **(d)** 10 TCID_50_. Changes in VNA titers depending on virus dose allow for evaluation of the test’s sensitivity and specificity. The dashed line with a black circle represents the antibody titers of individual rabbits, while the red thick line with a black square indicates the mean antibody titers in rabbit sera. (*) Titers are presented as reciprocal serum dilutions. **(e)** The diagram displays 95% confidence intervals for various virus doses applied in the neutralization test. Confidence intervals were calculated using two-way analysis of variance (2-way ANOVA), which statistically substantiated the selection of the virus concentration that provides the greatest difference between experimental groups and the highest measurement reliability.

Serum samples collected from rabbits vaccinated against cowpox virus on days 21 and 28 post-vaccination demonstrated the ability to neutralize virus doses of 50 and 100 TCID_50_ at titers up to 1:4. In contrast, serum samples obtained on day 14 did not exhibit neutralizing activity at these virus concentrations. However, when lower virus doses (25 and 10 TCID_50_) were used, the same sera showed neutralizing titers of 1:16 and higher, which were significantly greater than those observed with higher virus doses (50 and 100 TCID_50_) (**Figure 1**).

Multiple comparisons revealed statistically significant differences between the dose groups (p < 0.05), and all estimates were accompanied by 95% confidence intervals. These findings suggest that low virus doses are more sensitive for quantitatively evaluating neutralizing antibodies in the VNT. Based on the obtained results, a virus dose of 25 TCID_50_ was selected as the standard for evaluating the humoral immune response in rabbits vaccinated against cowpox virus in order to improve the sensitivity of the VNT.

### Titration of the challenge virus

Prior to evaluating the protective efficacy of the vaccine, the infectious dose (ID_50_) of the challenge virus was determined. The pathogenicity of the virus was assessed based on the appearance of cutaneous reactions – specifically, necrosis – at the sites of virus administration. The titration protocol for the “Cowpox-CAM” strain of CPXV in rabbits is presented in **Table 1**.

**Table 1:** Titer of the CPXV in rabbits.

Virus dilution	Rabbit 1	Rabbit 2	Rabbit 3	Rabbit 4
10^-1^	+	+	+	+
10^-2^	+	+	–	–
10^-3^	–	–	–	–
10^-4^	–	–	–	–
10^-5^	–	–	–	–
10^-6^				

(+) – indicates rabbits with necrosis at the site of virus inoculation.

(-) – indicates rabbits without skin lesions.

As shown in **Table 1**, all rabbits inoculated with the undiluted virus and the 10^-^¹ dilution developed necrotic lesions at the sites of virus administration. In the group infected with the 10^-^² dilution, necrosis was observed in only two animals. Conversely, no visible skin changes were detected in rabbits inoculated with virus dilutions ranging from 10^-^³ to 10^-6^. Based on these observations, the ID_50_ value was determined using the Reed and Muench method (15), and the results are summarized in **Table 2**.

**Table 2:** Calculation of the ID_50_ of the “Cowpox-CAM” strain of CPXV in rabbits.

Virus dilution	Number of rabbits without necrosis	Number of rabbits with necrosis	Cumulative without necrosis	Cumulative with necrosis	Percentage of rabbits with skin necrosis
1	2	3	4	5	6
whole virus*	0	4	0	10	100
10-1	0	4	0	6	100
10-2	2	2	2	2	50
10-3	4	0	6	0	0
10-4	4	0	10	0	0
10-5	4	0	14	0	0
10-6	4	0	18	0	0

(*) – non-diluted virus suspension.

In the first column of **Table 2**, the virus dilutions are presented. The second and third columns indicate the number of rabbits without necrosis and with skin necrosis, respectively, at each corresponding virus dilution. In the fourth column, the number of rabbits without skin necrosis is shown as follows: for the undiluted virus, the value corresponds to the number of animals without necrosis (as per the second column); for the 10^-^¹ dilution, it includes the number of rabbits without necrosis at this dilution plus those from the previous (more concentrated) dilution, and so on. This calculation assumes that any rabbit remaining unaffected at a lower dilution would likewise remain unaffected at higher dilutions.

In the fifth column, the cumulative number of rabbits with necrosis is calculated in reverse: for the highest dilution (10^-6^), the value corresponds to the number of animals with necrosis from the third column; for the 10^-5^ dilution, it includes the sum of necrotic animals at this dilution and the subsequent higher dilution (10^-6^); for each preceding dilution, the value includes the number of rabbits with necrosis at that dilution and all higher (more diluted) ones.

The sixth column presents the percentage of affected animals at each dilution, calculated using the following Equation 1:


(1) $$Percentage\;of\;skin\;lesions=\frac{100*\text{Cumulative number of animals with skin necrosis }\left(\text{from column }5\right)}{\begin{array}{c} Total\;number\;of\;animals\;with\;and\;without\;skin\;necrosis\;\\ \left(based\;on\;columns\;4\;and\;5\right) \end{array}}$$

The percentage of skin lesions depending on the whole virus and its dilutions was calculated as follows:


$$Percentage\;of\;skin\;lesions\;with\;whoe\;virus=\frac{100*10\;}{10}=100;$$

$$Percentage\;of\;skin\;lesions\;at\;10^{-1}virus\;dilution=\frac{100*6\;}{6}=100;$$

$$Percentage\;of\;skin\;lesions\;at\;10^{-2}\;virus\;dilution=\frac{100*2\;}{4}=50;$$

$$Percentage\;of\;skin\;lesions\;at\;10^{-3\;}virus\;dilution=\frac{100*0\;}{6}=0;etc.$$

The extent of skin lesions is inversely proportional to the logarithm of the virus dilution. This proportionality (x) was calculated using the following Equation 2:


(2) $$x=\frac{\text{Percentage of skin lesions at the highest critical dose}-50\%}{\text{Percentage of skin lesions at the highest critical dose}-\text{Skin lesion percentage at the threshold dose}}$$

$$x=\frac{100-50}{100-0}=0,5$$

(3) $$\mathrm{lg}ID_{50}=\text{B}+\frac{b-50}{\text{b}-\text{a}}*lgd,$$

*Where*:

B – the dilution giving an effect greater than 50%;

b – the percentage corresponding to dilution B;

a – the percentage corresponding to the dilution giving an effect less than 50%;

d – the dilution factor (e.g., 10 for tenfold dilution).

Substituting the obtained values into the Equation 3:


$$\mathrm{lg}ID_{50}=10{¯}^{2}+\frac{100-50}{100-0}*\left(-1\right)=\;-2+0,50*\left(-1\right)=-2,50.$$

Based on the obtained results, the infectious dose of the challenge virus was 10².^50^ ID_50_ per 0.1 mL, which corresponds to an absolute value of 316 ID_50_/0.1 mL.

The titer of the challenge Cowpox-CAM strain of CPXV was 10³.^50^ ID_50_ per 1 mL.

Thus, the analysis of the obtained results demonstrated that the appropriate dose for challenge infection of immunized rabbits with CPXV is 316 ID_50_ per 0.1 mL, which induces skin necrosis in 50% of infected animals. The ID_50_ was calculated using the Reed and Muench method and was found to correspond to a virus dilution of 10^-^² (1:100).

### Vaccine elicited immunity in rabbits assessed by protection against infection

Vaccine elicited immunity in rabbits (laboratory model) against cowpox virus challenge. The dose of the CPXV that induces skin lesions at the site of virulent virus administration in rabbits is of great importance, since this challenge infection of vaccinated and unvaccinated control animals with a pathogenic virus is considered a reliable method (gold standard) for evaluating the protective efficacy of the vaccine.

The vaccine was evaluated by CPXV infection of vaccinated and control (non-vaccinated) rabbits using intradermal administration of the previously determined infectious dose of the virulent virus (316 ID_50_/0,1 mL). Five rabbits were immunized subcutaneously with the vaccine at a dose of 10,000 TCID_50_ and five rabbits from the control group (injected with physiological saline) were challenged on day 21 post-vaccination with the virulent “Cowpox-CAM” strain of CPXV at a dose of 316 ID_50_.

**Figure 2:**
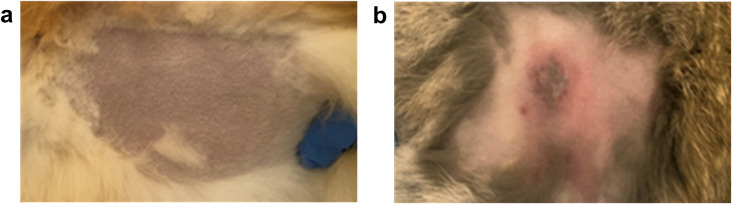
Clinical presentation of a vaccinated **(a)** and non-vaccinated **(b)** rabbit following challenge infection with the virulent “Cowpox-CAM” strain of CPXV.

The results demonstrated that the vaccinated rabbits (**Figure 2a**) remained alive and healthy without any clinical signs of disease throughout the observation period (21 days), whereas the non-vaccinated (control) rabbits developed illness with evident clinical symptoms, followed by generalization of the infectious process (**Figure 2b**).

Thus, the infectious dose of the virulent cowpox virus (316 ID_50_) was suitable for assessing the efficacy of the vaccine.

### Determination of the minimal antibody level following vaccination able to protect rabbits from the virulent virus

To determine the minimal level of antibodies following vaccination able to protect rabbits from virulent CPXV infection, 20 rabbits were immunized subcutaneously with the cowpox vaccine at a dose of 10,000 TCID_50_, and 3 rabbits (controls) were injected subcutaneously with 1 mL of Phosphate-Buffered Saline (PBS). On day 21 after vaccination, blood serum samples were collected from the rabbits and tested in a VNT using 25 TCID_50_ of the virus.

As a result of the study, it was established that the antibody titers in the sera of vaccinated rabbits ranged from 1:2 to 1:64, depending on individual immune responses. These data were compared with the clinical signs observed in the vaccinated rabbits following challenge infection at a dose of 316 ID_50_. The results of the analysis are presented in **Table 3**.

**Table 3:** Antibody levels and clinical signs in rabbits vaccinated against cowpox on day 21 post-vaccination.

Number of rabbits	Dose of vaccine virus	Antibody titer	Dose of challenge virus	Clinical signs of cowpox after challenge infection
1 rabbit	10,000 TCID_50_	1:2	316 ID_50_	Skin necrosis, death
1 rabbit		1:4		Skin necrosis
2 rabbits		1:8		Skin necrosis
6 rabbits		1:16		No changes
4 rabbits		1:32		No changes
6 rabbits		1:64		No changes
3 rabbits (control)	1 mL PBS	1:0		Hyperthermia, hyperemia at injection sites, skin necrosis, death

The data presented in **Table 3** indicate that, on day 21 post-vaccination, the antibody titer providing protection against the virulent CPXV at a dose of 316 ID_50_ in rabbits was 1:16 or higher. In this experiment, the proportion of resistant rabbits with antibody titers exceeding 1:16 was 80%.

Thus, the experiment assessing antibody levels in rabbits vaccinated against cowpox demonstrated that animals with antibody titers of 1:16 or higher were protected against a virulent strain of cowpox virus at a dose of 316 ID_50_.

## Discussion

Infections caused by orthopoxviruses represent an important model for studying the immunity. With pox viruses, cellular immunity plays a critical role in protection together with humoral immunity. This is demonstrated in animals that recover from experimental infection which have low levels of specific antibodies (17, 18).

These observations raise questions regarding the accuracy of diagnostic tests such as the virus neutralization test. The principle of this assay is based on the binding of antibodies to virions, which blocks key steps in viral replication and reduces or neutralizes its infectivity (12).

The VNT can be performed in two formats: for the detection of either antibodies or antigens. The method can be carried out in macro-format (in test tubes, on laboratory animals, or chicken embryos) or in micro-format (on cell culture plates). The micromethod was developed in 1990 (19). However, performing the neutralization assay is associated with certain challenges, including the requirement to work with live viruses and the need to comply with biosafety regulations for BSL-4, BSL-3, or enhanced BSL-2 laboratories (depending on the pathogen classification).

There are numerous protocols for conducting VNT, with parameters varying depending on the nature and structure of the virus. These parameters include the cell type used, the number of cells, seeding conditions, virus dose, and incubation time of the virus–serum–cell complex, and methods for evaluating the results.

The aim of this study was to determine the optimal virus dose for performing the VNT to assess the antibody titer in the serum from vaccinated rabbits against CPXV. Determine the optimal viral dose used to evaluate vaccine efficacy. As well as to determine the protective efficacy of the vaccine and to establish the minimum protective antibody level in vaccinated rabbits.

VNTs are typically performed using a standard virus dose ranging from 100 to 200 TCID_50_. However, previous studies have shown that the use of a 100 TCID_50_ virus dose does not always yield reproducible results (17, 18). Microbes, their antigens, as well as vaccine preparations act as irritants that, upon entering the internal environment, can induce a pathological process and elicit a defensive response aimed at restoring homeostasis (20). This study focused on evaluating antibodies as a protective response of the organism in response to the administration of a cowpox vaccine. Therefore, the VNT was optimized for sensitivity by titration of the virus dose. The optimized VNT was used to determine the minimal level of humoral antibodies able to provide protection against CPXV.

During the optimization of the virus dose for the VNT, doses of 100, 50, 25, and 10 TCID_50_ were used. The results demonstrated that at higher doses (100 and 50 TCID_50_), the neutralizing activity of the sera was low, not exceeding 2 log_2_. In contrast, when the virus dose was reduced to 25 and 10 TCID_50_, the neutralizing activity of the same sera increased to 4–6 log_2_. These findings are consistent with other studies where a virus dose of 10 TCID_50_ was used to assess the immunogenicity of vaccines (21–23).

In addition, similar results were observed in the analysis of sera from patients who had recovered from COVID-19. At a dose of 25 TCID_50_, higher neutralizing activity was noted compared to the standard dose of 100 TCID_50_ (24). A comparable approach was also applied in studies of sera from patients with monkeypox and those vaccinated with vaccinia virus (25).

Our findings confirm that the use of low virus doses (10–25 TCID_50_) in VNT enables the detection of humoral immunity in vaccinated animals alongside cellular immunity. The optimized VNT increases the sensitivity of the VNT without changing the specificity, thereby contributing to a more accurate assessment of the presence and level of neutralizing antibodies.

Thus, the identified optimal virus dose may be used in the future for the standardization of the VNT in the evaluation of vaccine immunogenicity against orthopoxviruses, facilitating more precise detection and quantification of neutralizing antibodies.

To evaluate the protective efficacy of vaccine preparations, a challenge test using a virulent virus is performed. The dose of the control CPXV that induces skin lesions at the site of inoculation in 50% of animals is of critical importance to ensure that the vaccine evaluation is optimized. To determine the 50% infectious dose (ID_50_) of the challenge virus, rabbits were intradermally infected with serial tenfold dilutions (from 10^-^¹ to 10^-6^) of the Cowpox-CAM strain of CPXV. The calculation of the ID_50_ revealed that the 50% infectious dose of the control virus for rabbits is 316 ID_50_ per 0.1 mL.

The use of the established control virus dose (316 ID_50_/0.1 mL) to assess the protective efficacy of the vaccine demonstrated that rabbits (n = 5) immunized subcutaneously with a dose of 10,000 TCID_50_ of the vaccine remained alive and clinically healthy 21 days post-immunization upon challenge with the virulent virus. In contrast, the control (non-vaccinated) rabbits (n = 5) developed disease with necrosis at the site of virus inoculation. These findings indicate that the vaccine’s immunogenic properties can be reliably evaluated using this laboratory model, which provides a basis for standardizing methods for vaccine potency testing against CPXV.

The next stage of our study was to determine the level of VNA that protect immunized rabbits from the virulent CPXV. Serum samples from rabbits immunized subcutaneously with 10,000 TCID_50_ of the cowpox vaccine were examined on day 21 post-immunization using the SNT with a virus dose of 25 TCID_50_. The results showed that the VNA titers in rabbit sera ranged from 1:2 to 1:64, depending on individual variability among the vaccinated animals. Upon challenge with 316 ID_50_ of the virulent virus, a protective antibody level was determined to be a titer of 1:16 or higher. Based on the outcomes of the challenge test, 80% of the rabbits were considered immune to CPXV. These findings correlated with the absence or presence of clinical signs observed in the vaccinated rabbits following challenge with the virulent virus at a dose of 316 ID_50_.

Our results are consistent with the findings of other authors (26), who reported that a neutralizing antibody titer of at least 1:20 in human serum is considered sufficient for protection against smallpox following vaccination with vaccinia virus.

Moreover, the protective antibody threshold varies depending on the type of infection. For instance, in the case of rabies, the antibody titer in the serum of vaccinated animals should be no less than 1:64 (corresponding to a VNA level of at least 0.5 IU/mL), as determined by the biological neutralization test in white mice, indicating effective vaccine-induced protection (27). Similarly, other researchers (28) have suggested that in birds vaccinated with live vaccines against Newcastle disease, the antibody titer should range between 3 and 4 log_2_, which corresponds to titers of 1:8 to 1:16.

These results further underscore the importance of establishing standardized protective antibody thresholds across orthopoxvirus vaccines. Recent studies have underscored the central role of neutralizing antibodies as correlates of protection against orthopoxviruses. For instance, Edghill-Smith et al. (2005) experimentally demonstrated that smallpox vaccine–induced antibodies were both necessary and sufficient to protect nonhuman primates from monkeypox virus infection (29). These findings align with our results, suggesting that the robust neutralizing titers elicited by cowpox vaccination in rabbits represent an effective correlate of protection. Importantly, this supports the rationale for setting minimal antibody thresholds in vaccine potency testing.

Further, cross-reactivity studies have shown that sera from individuals vaccinated against smallpox exhibit strong neutralizing activity against monkeypox and vaccinia viruses, targeting conserved antigens such as A35R, A33R, B5R, L1, and D8 (30). These antigens are shared across orthopoxviruses, indicating that vaccine-induced antibodies can confer broad protection within this viral genus.

Our data also gain relevance when compared to international benchmarks. For example, the MVA-BN vaccine (JYNNEOS/Imvamune), approved by both FDA and WHO for monkeypox prevention, has shown protective efficacy in preclinical studies, but recent findings indicate low and short-lived antibody responses in vaccinia-naïve individuals (31). This underscores the importance of quantitative serological evaluation and highlights the utility of our optimized rabbit model for establishing protective thresholds against cowpox virus.

## Conclusion

In the study, the use of 25 TCID_50_ of virus in the VNT enables the detection of antibody levels in vaccinated or convalescent animals. In rabbits immunized with the cowpox vaccine, an antibody titer of 1:16 or higher provides protection against challenge with 316 TCID_50_/mL of the virulent CPXV. The obtained results support the reliable evaluation of the immunogenic properties of the cowpox vaccine using a laboratory model and contribute to the standardization of vaccine potency control methods.

## Data Availability

The original contributions presented in the study are included in the article/supplementary material. Further inquiries can be directed to the corresponding author.
